# Depression and loneliness of older adults in Europe and Israel after the first wave of covid-19

**DOI:** 10.1007/s10433-021-00640-8

**Published:** 2021-08-24

**Authors:** Josefine Atzendorf, Stefan Gruber

**Affiliations:** Max Planck Institute for Social Law and Social Policy, Munich Center for the Economics of Aging (MEA), Amalienstr. 33, 80799 Munich, Germany

**Keywords:** Loneliness, Sadness/depression, Covid-19, Cross-country comparison, Multilevel analysis, SHARE

## Abstract

Epidemic control measures that aim to introduce social distancing help to decelerate the spread of the COVID-19 pandemic. However, their consequences in terms of mental well-being might be negative, especially for older adults. While existing studies mainly focus on the time during the first lockdown, we look at the weeks afterward in order to measure the medium-term consequences of the first wave of the pandemic. Using data from the SHARE Corona Survey, we include retired respondents aged 60 and above from 25 European countries plus Israel. Combining SHARE data with macro-data from the Oxford COVID-19 Government Response Tracker allows us to include macro-indicators at the country level, namely the number of deaths per 100,000 and the number of days with stringent epidemic control measures, in addition to individual characteristics. The findings show that both macro-indicators are influential for increased feelings of sadness/depression, but that individual factors are crucial for explaining increased feelings of loneliness in the time after the first lockdown. Models with interaction terms reveal that the included macro-indicators have negative well-being consequences, particularly for the oldest survey participants. Additionally, the results reveal that especially those living alone had a higher risk for increased loneliness in the time after the first COVID-19 wave.

**Supplementary Information:**

The online version contains supplementary material available at 10.1007/s10433-021-00640-8.

## Introduction

As a reaction to the sudden emergence of COVID-19, most countries implemented epidemic control measures that aimed at ‘social distancing.’ In order to decelerate the spread of the pandemic, those policies urged people to keep physical distance to others, to reduce social contacts to a minimum, and to avoid leaving their places of residence for activities deemed unnecessary. Additionally, group activities were prohibited in many countries and a large number of public facilities were closed. One of the central aims of those measures was protecting older people against an infection as they represent the population group most at risk for severe disease progression and possible death related to or directly from a COVID-19 infection (Posch et al. 2020). Therefore, contact with older people was explicitly discouraged.

While social distancing showed positive effects regarding infection numbers (Voko and Pitter 2020), the socio-psychological consequences are quite discouraging. Social isolation increases the risk for mental health problems (Santini et al. 2020). Policy measures that call for social distancing might therefore directly affect feelings of loneliness and depression (Armitage and Nellums 2020). In particular, seniors and individuals living alone, who seem even more vulnerable to mental health problems, could be affected by those developments (Bae 2020; Chou et al. 2006; Dykstra et al. 2005). Mental health problems themselves are known to be associated with an increasing risk for morbidity and mortality in the long term, especially for older people (Holt-Lunstad et al. 2015). Moreover, social isolation in older people has been shown to lead to elevated risks of cardiovascular, autoimmune, and neurocognitive diseases (Gerst-Emerson and Jayawardhana 2015).

A large number of studies analyzing loneliness and depression during the first lockdown have been published. Their findings depend on region and age group under study. Krendl and Perry (2020) focus on older adults in the US. They find that their mental health was negatively affected by the COVID-19 pandemic. The respondents experienced higher levels of depression and loneliness than they had prior to the pandemic. These results are consistent with findings on young and middle-aged adults in the US (Killgore et al. 2020) and in China (Wang et al. 2020). Van Tilburg et al. (2020) find that the loneliness level of older people in the Netherlands increased while mental health in general remained roughly stable. The study shows that social distancing measures were not the main driving factor for the increased loneliness prevalence. Instead, personal losses, worries about the pandemic, and a decline of trust in societal institutions were associated with increased mental health problems and feelings of loneliness. For the general population in the UK, Groarke et al. (2020) find that rates of loneliness during the initial phase of the lockdown were high, but that the risk factors were not specific to the COVID-19 pandemic. The authors conclude from group-specific analyses that supportive interventions to reduce loneliness should focus on younger people and those with mental health symptoms.

Although important insights were gained from the existing research about feelings of loneliness and depression during the first lockdown, some important questions remain unanswered. The first one concerns the timing. While existing studies mainly focus on the time during the lockdown itself, we look at the weeks afterward in order to measure the medium-term consequences of the first wave and the lockdown measures on the mental well-being of older people. The second research gap addressed by our study is that existing findings refer to one single country. Country comparative analyses, however, can shed light on the question of whether the prevalence of loneliness and depression differs between countries and which macro-level factors may explain those possible differences. This is directly connected to a third research gap in existing studies: the inclusion of macro-indicators. By including data from the Oxford COVID-19 Government Response Tracker (OxCGRT), we examine whether mental health consequences in the time after the lockdown are associated with the national epidemic control policies and with the general pandemic circumstances.

## Theoretical considerations and hypotheses

The focus of our study is on the retired population aged 60 and above who might be particularly affected by the pandemic, not only in terms of disease progression in case of an infection, but in terms of loneliness and depression. Loneliness can be defined as the unpleasant state of acknowledging a discrepancy between the desired amount of contacts or emotional support and that available in one’s environment (Perlman and Peplau 1981). Loneliness is characterized by a perceived lack of control over the quantity and the quality of one’s social activity (Luhmann and Hawkley 2016). The pandemic and the social distancing measures were additional factors that limited an individual’s control over social contacts and activities, a circumstance, which could have especially affected older parts of the population. Longitudinal and life-course analyses suggest a decline in the size of social networks with increasing age (Kalmijn 2012). McDonald & Mair (2010) show that not only the network size but also the number of daily social interactions is negatively associated with age. Additionally, Zhaoyang et al. (2018) show that those daily interactions, while fewer in number, are more valued in later life. The reduction in social contacts as a consequence of the pandemic might have hit those at the margins of the age distribution particularly hard, possibly leading to increased feelings of loneliness and depression.

The consequences of the lockdown might not only be notable at the individual level. The first wave of the pandemic hit countries and regions with both different timings and intensities. While, for example, Greece had moderate case numbers at the beginning and throughout the first wave, Israel and some regions in Italy had very high case numbers even at a very early stage of the first wave (Last 2020). Additionally, the national epidemic control policies differed as well. While some countries introduced a strict lockdown of lengthy duration, others implemented less strict measures for only a short period. One exception is Sweden, which did not implement compulsory measures at all during the first wave. Instead, the Swedish approach was characterized by recommendations on a voluntary basis, e.g., regarding good hand hygiene, mindfulness of physical distance, and refraining from large gatherings and non-essential travel (Kavaliunas et al. 2020). We expect those differences at the country level to be reflected in the levels of loneliness and depression. Kim and Jung (2020) showed that distress associated with the pandemic correlates with the stringency of the implemented policy measures. Furthermore, the correlation of distress and social distancing is moderated by the number of deaths related to COVID-19 (Kim and Jung 2020). Based on these findings and considerations, we formulate our first hypothesis in two steps as follows:

*H1a*: Number of deceased and the stringency of policy implications account for country variation in depression and loneliness.

*H1b*: Both the general situation of the pandemic, approximated by the number of deceased, and the duration of stringent policy measures have a significant influence on older people’s well-being at the micro-level.

The prevalence of loneliness and depression varies between age groups. While depression is less prevalent among older adults than among younger adults, suicide rates are higher than in younger adults and more closely associated with depression (Fiske et al. 2009). The prevalence of loneliness turns out to be higher among older people (Dykstra 2009). Here, according to Weiss (1973), two types of loneliness need to be distinguished: emotional loneliness and social loneliness. While emotional loneliness is the absence of intimate or close emotional attachment, social loneliness originates from the absence of a broader group of contacts or the engagement in social networks (Dykstra 2009). The risk for social loneliness might be higher for younger adults, whereas the risk for emotional loneliness as a consequence of the lockdown might be higher for older persons. In their study on older adults in the Netherlands, Tilburg et al. (2020) showed that average social loneliness increased only slightly during the lockdown, while average emotional loneliness increased strongly. The age differences in loneliness may arise from (i) risk factors associated with loneliness being more prevalent in one age group than in another, or (ii) that the relative impact of a specific risk factor varies between age groups. Previous research identified the living arrangements as one important factor (Luhmann and Hawkley 2016). In particular, older unmarried persons living alone might be particularly affected by the lockdown. Statistics reveal a U-shaped distribution. As Luhmann and Hawkley (2016) showed for the German context, among both younger and older adults, the proportion of people living alone is substantially higher than among middle-aged adults. Our second hypothesis is based on these considerations and addresses effect-heterogeneity within the older population:

*H2*: The oldest age group and those living in single households have an increased risk of intensified feelings of depression and loneliness after the first COVID-19 wave.

Our last hypothesis refers to factors that might help to reduce the risk of intensified feelings of loneliness and depression, especially in pandemic times. Here we set the focus on direct and electronic contacts. While new media are still considered to strengthen processes which induce the feeling of loneliness in young adults, it has been argued that the Internet and other communication tools, such as social network sites, may have the potential to become instruments in the fight against loneliness in older individuals (Fokkema and Knipscheer 2007). We follow the first findings in this research area. Fingerman et al. (2020) suggest that in-person contact may confer distinct benefits not available via electronic contacts. Krendl and Perry (2020) find that electronic communication did not offset older adults’ loneliness. Therefore, our last hypothesis is as follows:

*H3*: Electronic contacts do not significantly reduce the risk of feeling more depressed or lonely as a consequence of the pandemic.

### Data and methods

#### Database and sample

We used the Preliminary Wave 8 Release 0 data set of the SHARE Corona Survey Börsch-Supan (2020) that was conducted from June to August 2020 via computer-assisted telephone interviews (CATI) (Scherpenzeel et al. 2020). We further utilized the OxCGRT (2020) for aggregated data on COVID-19 related death figures and the duration of stringent measures on the country level (Hale et al. 2020).

A total of 51,478 respondents participated in the SHARE Corona Survey. We excluded non-retired participants (*n* = 13,741) and those younger than 60 (*n* = 6,321) irrespective of their employment status which leads to a considerable reduction in the sample size. The reason behind this decision is that the well-being consequences of the pandemic and the epidemic control measures may vary between younger respondents still working and older retired respondents. Working respondents may be economically more affected by the pandemic. Moreover, as not all employers are equally affected in economic terms, there would be additional unobserved heterogeneity when including working respondents. In addition, social networks and contacts outside the household differ between age groups and between working and retired, which may again lead to different implications for loneliness and depression. Therefore, the focus on retired respondents aged 60 plus considerably reduces heterogeneity and allows specific analyses for this group.

Furthermore, we excluded respondents from Malta (*n* = 347), because at the time when we accessed the OxCGRT data, it did not contain aggregated data for Malta. Therefore, the analytical sample contains data from 26 countries: Germany, Sweden, the Netherlands, Spain, Italy, France, Denmark, Greece, Switzerland, Belgium, Czech Republic, Poland, Portugal, Luxembourg, Hungary, Slovenia, Estonia, Croatia, Lithuania, Bulgaria, Cyprus, Finland, Latvia, Romania, and Slovakia, plus Israel as the only non-European country. After the exclusion of respondents due to item non-response, the final analytical sample contains 27,889 participants for cross-sectional analyses based on the SHARE Corona Survey.

### Measurements of loneliness

In the SHARE Corona interview, a change in loneliness is assessed by asking the respondents who answered that they felt lonely often or some of the time whether they felt lonelier, less lonely, or about the same compared to the time before the outbreak of COVID-19. This question is categorized into a binary variable where 1 is defined as *feeling lonelier* and 0 as *feeling less lonely or about the same* as before the outbreak. Respondents who answered that they did not feel lonely are coded as zero; otherwise, they would be missing.

### Measurements of depression

Changes in feelings of sadness/depression are assessed by asking respondents who have been sad or depressed in the past month whether they felt less, more, or about the same sad/depressed compared to the time before the outbreak of the pandemic. We transformed this variable into a binary indicator that equals 1 for those *feeling more sad or depressed* and zero for the ones feeling less sad or depressed or about the same. Again, respondents who answered that they did not feel sad or depressive in the last month are coded as zero.

### Exploratory variables at individual level

Age is grouped into three categories: 60 to 69 years old, 70 to 79 years old, and older than 80 years. Household size is categorized into a dichotomous variable with 1 indicating *individuals living alone* and 0 indicating *individuals living together with at least one additional person*. The frequency of social contacts is assessed for personal social contacts and electronic social contacts. Respondents are asked how often they had personal social contacts (face-to-face) or electronic contact (over phone, e-mail, or other electronic means) with their children, parents, other relatives, and non-relatives since the outbreak. Possible answers are *daily*, *several times a week*, *about once a week*, *less often*, or *never*. We recoded the variables for personal and electronic contacts into two variables with two categories each: *Less than weekly* or *at least once a week*.

### Exploratory variables at macro-level

At the country level, we used the number of days with stringent measures and the number of cumulated deaths related to COVID-19 per 100,000 inhabitants for the time the pandemic started until the end of the field phase. The stringency index provided by OxCGRT combines the stringency of political indicators and public information campaigns (Hale et al. 2020; OxCGRT 2020). Political indicators include school closings, workplace closings, canceling of public events, restrictions on gathering size, closing public transport, stay-at-home requirements, restrictions on local movement, and restrictions on international traveling. The stringency index ranges in values from 0 to 100, with 0 being the least stringent. For a detailed description of the stringency index, please see OxCGRT (2020).

### Control variables

We included whether the respondents’ health is worse, improved, or unchanged compared to before the outbreak of the pandemic. Again, we used a binary variable, with 1 indicating a *worsened health status* and 0 indicating that the *health status improved or is about the same*. Regarding a COVID-19 infection, participants were asked whether they themselves or someone close to them had been infected, hospitalized, or had died due to COVID-19. As additional characteristics, we included gender, education, marital status, and financial hardship. In order to compare the internationally diverse educational degrees, SHARE contains the international standard classification of education (ISCED) (UNESCO 2011). We group the ISCED levels into three categories: *low*, *medium*, and *high* level of education. The marital status of respondents can be *married, never married, divorced*, and *widowed*. Financial problems are assessed by asking the respondents if the household has been able to make ends meet since the outbreak of COVID-19. We recoded this question into a binary variable with 1 indicating that the household is *making ends meet with great* or *some difficult*y and 0 that the household is *making ends meet fairly easily* or *easily*. If individuals did not answer this question, we used information about making ends meet from SHARE Wave 8 data collected before the outbreak of the pandemic.

### Analytical strategy

In order to examine which factors influence post-lockdown loneliness and feelings of sadness/depression, we applied multilevel binary logistic regression models with two levels (individual and country level). Feeling more depressed and feeling lonelier after the outbreak of COVID-19 were each used as separate dependent variables. Multilevel analyses are needed to adjust standard errors, which are likely to be biased if the hierarchical structure of the data is ignored, because regression analyses are based on the assumption of independent residuals (Field 2013; Hox 2010; Raudenbush and Bryk 2002). To assess the fit of the model, we used the Akaike Information Criterion (AIC) and the Bayesian Information Criterion (BIC). For both criterions, a smaller value indicates a better model fit. To measure the effect of heterogeneity between countries, the Median Odds Ratio (MOR) was used. A value above one in the MOR indicates that living in a certain country may explain the variance in loneliness or depression (Merlo et al. 2006).

In a first step, no predictors were included in the multilevel models to estimate whether the prevalence of loneliness and depression varies between countries (intercept-only model). Significant variance components and a MOR greater than one indicate differences across countries. In the next model, predictors at the individual level and the country level were included as fixed effects with random intercepts to estimate the direct associations between the predictors and feelings of depression/loneliness. The direct effect of the predictors at country level on the outcomes were assessed by multiplying the logit of the odds ratio with 30 (for the increase in the outcome for 30 days with high stringency measures) or 50 (for the increase in the outcome if the number of deaths due to COVID-19 increases by 50 in 100,000 inhabitants). Furthermore, in the final model we calculated average marginal effects (AME) to estimate interactions of the exploratory variables with the variables at the macro-level. As the models assume that the random effects are uncorrelated with the regressors, we also run models with fixed country level intercepts (fixed effects) as a robustness check.

All analyses were performed using Stata 14 SE (Stata Corp LP, College Station, TX). List-wise deletion was introduced for cases with missing information. There were no signs of multicollinearity among the predictors (Tolerance > 0.2; Variance Inflation Factor < 10). The data was weighted using cross-sectional calibrated weights as provided by SHARE.

### Results

#### Descriptive analyses

Table 1 summarizes the descriptive statistics of the analytical sample. The sample comprises 27,889 retired respondents above the age of 60 with a mean age of 74.5 years and a share of 56 percent female respondents. Table 2 shows the number of respondents per country. 4497 of the sample answered with ‘yes’ to the question whether they felt sadder/more depressed compared to the time before the pandemic. This seems to be a moderate share of respondents with 16.1 percent. However, excluding those who reported no feelings of sadness/depression, the share of respondents who reported an increase in sad/depressed feelings makes up more than 60 percent of sad/depressed respondents. The share of those who reported an increase in loneliness makes up 12.3 percent of the overall sample and 40 percent of those with feelings of loneliness.

**Table 1:** Descriptive statistics, data: preliminary SHARE wave 8 release 0

	*n*	Percent
Feeling more depressed than before outbreak	4,497	16.1
Feeling lonelier than before outbreak	3,436	12.3
*Age group*		
60 to 69	8,036	28.8
70 to 79	12,784	45.8
80 +	7,069	25.4
Female	15,626	56.0
*Education*		
Low	10,015	35.9
Medium	11,696	41.9
High	6,178	22.2
*Marital status*		
Married or registered partnership	17,850	64.0
Never married	1,241	4.5
Divorced	2,279	8.2
Widowed	6,519	23.4
Single household	8,581	30.8
Making ends meet with some or great difficulty	9,591	34.4
Worse health status than before outbreak	2,655	9.5
Respondent or anyone close tested positive	1,741	6.2
Hospitalization of respondent or anyone close due to Covid-19 infection	917	3.3
Anyone close died due to Covid-19 infection	732	2.6
Personal contact at least once a week	8,467	30.4
Electronic contact at least once a week	15,154	54.3
Total	27,889

**Table 2:** Number of respondents per country, data: preliminary SHARE wave 8 release 0

Country	Number of respondents
Cyprus	365
Netherlands	396
Portugal	407
Slovakia	481
Bulgaria	487
Luxembourg	500
Latvia	520
Israel	525
Hungary	624
Lithuania	675
Finland	798
Spain	815
Romania	893
Sweden	979
Croatia	1,028
Denmark	1,051
Switzerland	1,181
France	1,433
Poland	1,483
Italy	1,561
Greece	1,580
Germany	1,634
Belgium	1,938
Czech Republic	2,019
Slovenia	2,151
Estonia	2,365
Total	27,889

**Fig. 1:**
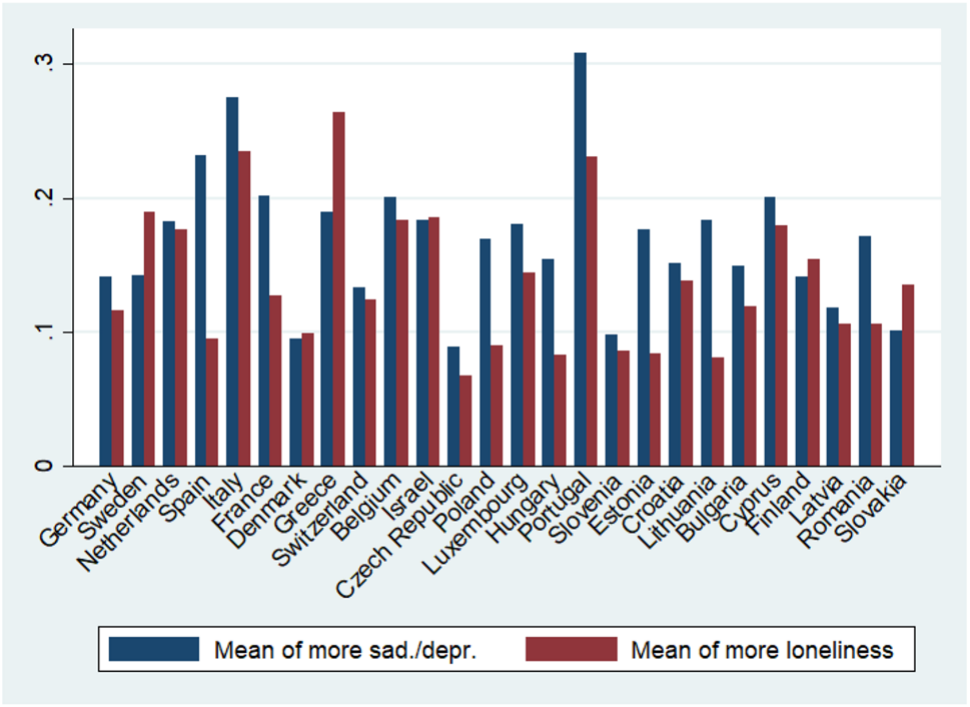
Country differences in the prevalence of more sadness/depression and more loneliness, Data: Preliminary SHARE Wave 8 Release 0 (*n* = 27,889)

Plotting the country specific means of increased feelings of sadness/depression and loneliness after the outbreak of the pandemic reveals a large variation between countries. As Fig. 1 illustrates, the share of respondents reporting increased feelings of sadness/depression (blue bars) ranges between more than 30 percent in Portugal and less than 10 percent of Danish, Czech, and Slovenian respondents. With regard to loneliness (red bars), more than 20 percent of Greek and Italian respondents reported feeling more lonely, whereas this is the case for 5 percent of Hungarian and 7 percent of Czech respondents.

**Fig. 2:**
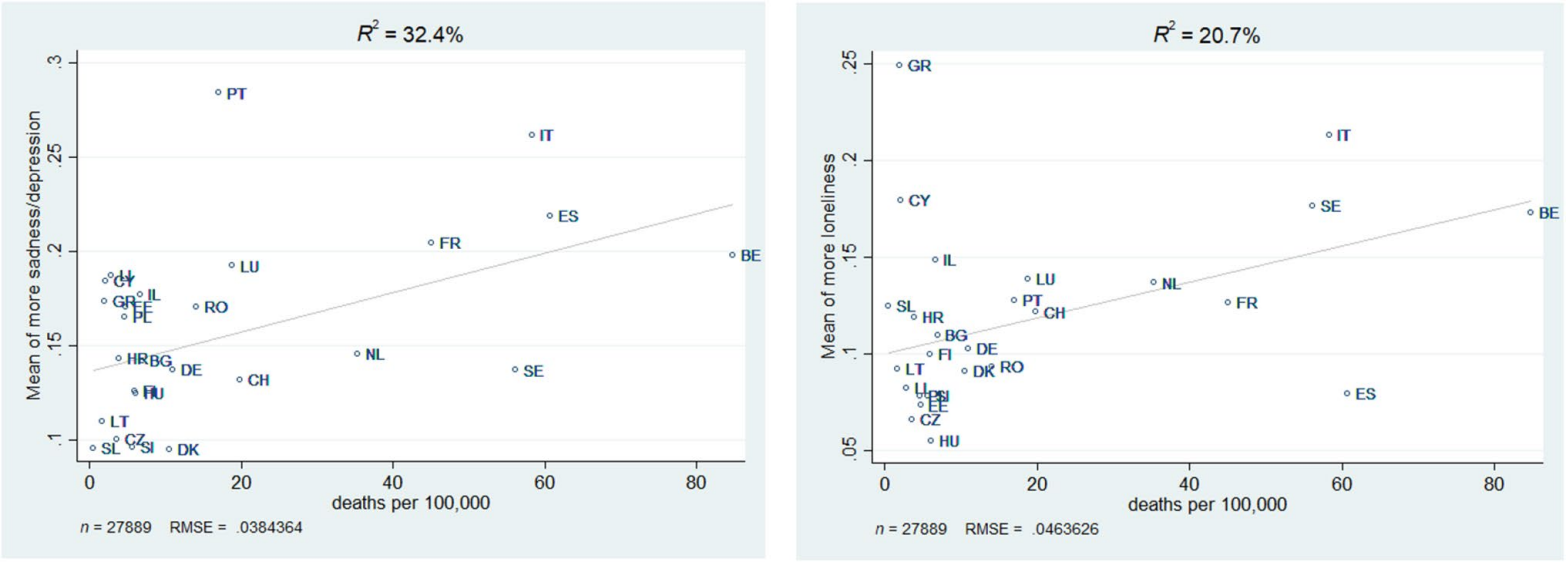
Association between country differences in the prevalence of more sadness/depression and loneliness with deaths per 100,000, Data: Preliminary SHARE Wave 8 Release 0 (*n* = 27,889)

**Fig. 3:**
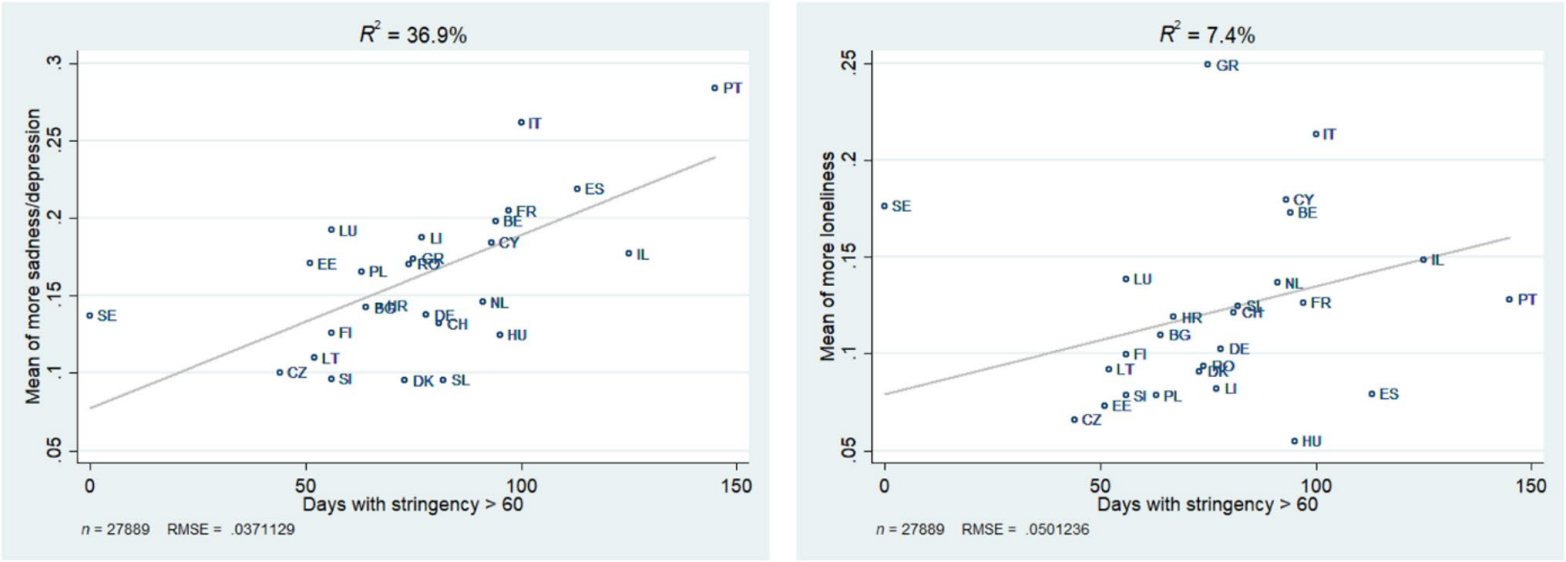
Association between country differences in the prevalence of more sadness/depression and loneliness with days with stringency index above 60, Data: Preliminary SHARE Wave 8 Release 0 (*n* = 27,889)

In order to test whether the differences between countries are associated with indicators on the country level, we plotted the country-specific means of both outcome measures against (i) the number of deaths per 100,000 population and (ii) the number of days with a stringency index above 60. As Figs. 2 and 3 show, the macro-indicators explain more of the country variance in sadness/depression than they do for increased feelings of loneliness. The number of deaths explains 32.4 percent of the country variance regarding the prevalence of more sadness/depression and 20.7 percent regarding the prevalence of being lonelier. The number of days with a stringency index above 60 explains 36.9 percent of the variance in sadness/depression and only 7.4 percent of the variance in loneliness.

### Testing for significant effects of individual-level and country-level predictors on feelings of increased depression since the outbreak of COVID-19 using multilevel logistic regression

#### Intercept-only model

As shown in Table 3, the variance component for the intercept-only model of increased feelings of sadness/depression is statistically significant, indicating that prevalence rates of feeling more depressed after the outbreak of COVID-19 differ between countries (MOR = 1.36, AIC = 26,005.53, BIC = 26,022).

**Table 3:** Multilevel analyses predicting feeling more depressed since the outbreak (*n* = 27,889), Data: Preliminary SHARE Wave 8 Release 0

	Intercept-only model		Fixed predictors at individual level with random intercepts		Fixed predictors at individual and macro-level with random intercepts	
	AIC = 26,005.53 BIC = 26,022		AIC = 24,034.43 BIC = 24,182.68		AIC = 23,997.79 BIC = 24,162.51	
Variables	OR	CI	OR	CI	OR	CI
Intercept	0.22***	(0.18–0.25)	0.10***	(0.08–0.11)	0.04***	(0.03–0.06)
Age						
*60–69 years*			*Ref*.		*Ref*.	
*70–79 years*			1.00	(0.90–1.10)	1.00	(0.90–1.11)
*> 80 years*			1.06	(0.95–1.20)	1.07	(0.94–1.21)
Living alone			1.20	(0.98–1.48)	1.20	(0.98–1.46)
Female			1.91***	(1.62–2.27)	1.92***	(1.63–2.27)
Education						
*Low education*			*Ref*.		*Ref*.	
*Medium education*			0.87**	(0.79–0.96)	0.87**	(0.79–0.96)
*High education*			0.92	(0.80–1.05)	0.92	(0.81–1.05)
Marital status						
*Married or registered partnership*			*Ref*.		*Ref*.	
*Never married*			0.87	(0.60–1.27)	0.87	(0.59–1.27)
*Divorced*			0.99	(0.83–1.19)	0.98	(0.82–1.18)
*Widowed*			0.98	(0.84–1.14)	0.98	(0.84–1.14)
Making ends meet			1.44***	(1.28–1.62)	1.46***	(1.31–1.64)
Worsened health status			4.62***	(3.82–5.59)	4.62***	(3.83–5.57)
Anyone tested positive for COVID-19			1.08	(0.89–1.31)	1.07	(0.88–1.30)
Anyone hospitalized due to COVID-19			0.96	(0.69 – 1.33)	0.96	(0.69 – 1.33)
Anyone died due to COVID-19			1.64***	(1.23–2.18)	1.62***	(1.22–2.16)
Personal contacts at least once a week			0.89*	(0.80–0.99)	0.89*	(0.80–0.99)
Electronic contacts at least once a week			1.14*	(1.01 – 1.28)	1.14*	(1.02–1.28)
Deaths per 100,000					1.01***	(1.01–1.01)
Days with stringency > 60					1.01***	(1.00–1.01)
	Var.	CI	Var.	CI	Var.	CI
Intercept	0.10	(0.06–0.20)	0.10	(0.06–0.17)	0.01	(0.00 –0.04)

AIC = Akaike Information Criterion, BIC = Bayesian Information Criterion, OR = odds ratio, CI = 95%-confidence interval, Var. = variance component,

*Ref.* = reference category, *** *p* < 0.001, ** *p* < 0.01, * *p* < 0.05

#### Fixed predictors with random intercepts

At the individual level, respondents with personal contacts at least once a week (OR = 0.89; 95%-KI = [0.80–0.99]) reported feeling less sad/depressed since the outbreak of the pandemic. Respondents with electronic contacts at least once a week (OR = 1.14; 95%-KI = [1.02–1.28]) were more likely to report more feelings of sadness or depression. Age and living alone are not significantly associated with the outcome variable. At the macro-level, the number of cumulated deaths (OR = 1.01; 95%-KI = [1.01–1.01]) and the number of days with stringent measures (OR = 1.01; 95%-KI = [1.00–1.01]) are significantly associated with feeling sadder or more depressed since the pandemic started. The model with fixed predictors and random intercepts at individual level improves compared to the intercept-only model (AIC = 24,034.43; BIC = 24,182.68) and when including the two macro-level indicators (AIC = 23,997.79, BIC = 24,162.51). Depression increases by 20% within 30 days with high stringency measures. If the number of deaths due to COVID-19 would increase by 50 in 100,000 inhabitants, feelings of depression and sadness would increase by 54%. When comparing the multilevel model with random intercepts at individual level with a model with fixed intercepts at individual level for checking robustness, we find small differences regarding the significance of living alone, personal contacts and medium education. There were no differences regarding the other predictors and control variables.

#### AMEs of interactions with exploratory variables at individual level with macro-variables

**Fig. 4:**
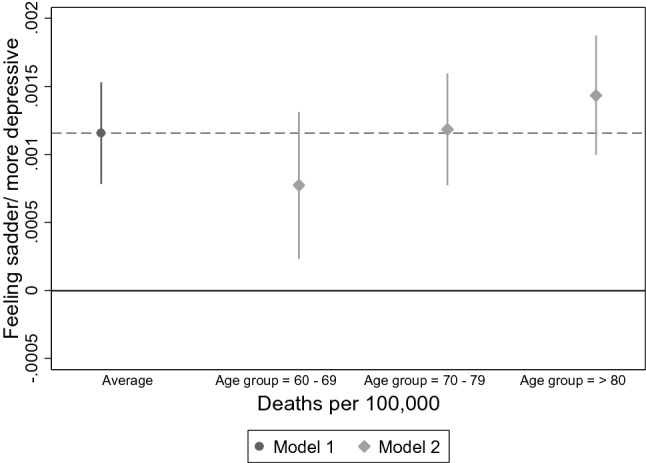
Average marginal effects of main and interaction effects for feeling sadder/more depressive and deaths per 100,000 for each age group (*n* = 27,889). Note. *Model 1* = *Main effect of deaths per 100,000, Model 2* = *interaction with number of deaths and age*. AMEs were calculated for the model with fixed predictors at individual and macro-level with random intercepts, Data: Preliminary SHARE Wave 8 Release 0

Figure 4 shows the average marginal effect for number of deaths on feeling sadder or more depressed, calculated from the model with fixed predictors at individual and macro-level with random intercepts ("Model 1"). Figure 4 shows the conditional effects for each age group, calculated from a model including an additional multiplicative interaction term of number of deaths with age ("Model 2"). Including interactions revealed that, while feelings of sadness or depression are increasing with number of deaths for all, the effect is significantly more pronounced in the oldest age group. In other words, the oldest old are the ones most likely to develop feelings of sadness or depression in countries with high mortality rates due to COVID-19.

**Fig. 5:**
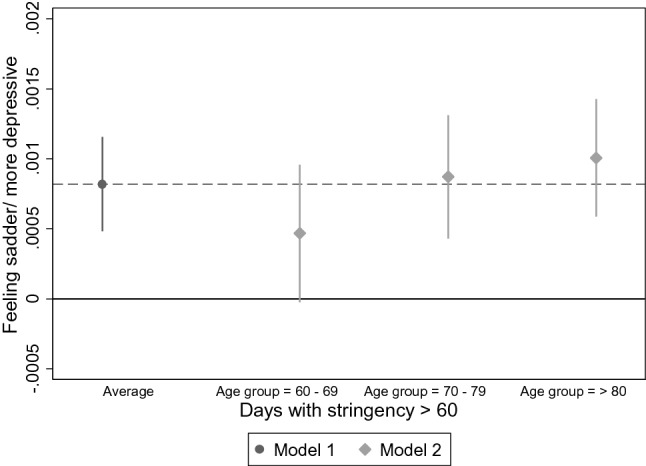
Average marginal effects of main and interaction effects for feeling sadder/more depressiv and number of days with stringency measures above 60 for each age group (*n* = 27,889). *Model 1* = *Main effect of number of days with stringency above 60 0, Model 2* = *interaction with number of days with stringency above 60 and age*. AMEs were calculated for the model with fixed predictors at individual and macro-level with random intercepts, Data: Preliminary SHARE Wave 8 Release 0

Figure 5 displays the average marginal effects for the number of days with a stringency index above 60. As with number of deaths, while feelings of sadness or depression are on average increasing with number of days with stringent measures, the effect is smaller for the youngest age group and not significantly different from zero. By tendency, effects are more pronounced and significant in the older age groups, but age group differences are not statistically significant. The inclusion of interactions between the macro-variables and living alone produces a similar pattern as for age, with respondents living alone reacting somewhat more sensitive (see Supplementary Material). However, interactions were not significant at the 5 percent level. Further, the effect of both macro-variables remained consistently positive, irrespective of frequency of personal or electronic contact, with no significant group differences emerging.

### Testing for significant effects of individual-level and country-level predictors on feeling lonelier since the outbreak of COVID-19 using multilevel logistic regression.

#### Intercept-only model

As seen in Table 4, the variance component for the intercept-only model of feeling lonelier is statistically significant, indicating that prevalence rates of feeling lonelier after the outbreak differ between countries (MOR = 1.42, AIC = 21,705.14, BIC = 21,721.61).

**Table 4:** Multilevel analyses predicting feeling lonelier since the outbreak of Covid-19 (*n* = 27,889), Data: Preliminary SHARE Wave 8 Release 0

	Intercept-only model		Fixed predictors at individual level with random intercepts		Fixed predictors at individual and macro-level with random intercepts	
	AIC = 21,705.14 BIC = 21,721.61		AIC = 19,914.72 BIC = 20,062.97		AIC = 19,912.82 BIC = 20,077.54	
BIC = 20077.						
Variables	OR	CI	OR	CI	OR	CI
Intercept	0.15***	(0.13–0.18)	0.05***	(0.04–0.07)	0.04***	(0.02–0.07)
Age						
*60–69 years*			*Ref*.		*Ref*.	
*70–79 years*			1.05	(0.89–1.23)	1.05	(0.89–1.23)
*> 80 years*			1.13	(0.99–1.30)	1.13	(0.99–1.30)
Living alone			2.19***	(1.72–2.80)	2.19***	(1.72–2.80)
Female			1.68***	(1.38–2.04)	1.68***	(1.39–2.04)
Education						
*Low education*			*Ref*.		*Ref*.	
*Medium education*			0.88*	(0.79–1.00)	0.89	(0.79–1.00)
*High education*			0.94	(0.83–1.07)	0.95	(0.84–1.07)
Marital status						
*Married or registered partnership*			*Ref*.		*Ref*.	
*Never married*			0.83	(0.54–1.26)	0.82	(0.54–1.26)
*Divorced*			1.14	(0.85–1.52)	1.13	(0.85–1.51)
*Widowed*			1.04	(0.87–1.25)	1.04	(0.87–1.25)
Making end meet			1.44***	(1.29–1.60)	1.44***	(1.29–1.61)
Worsened health status			3.49***	(3.09–3.96)	3.49***	(3.08–3.95)
Anyone tested positive for COVID-19			1.19*	(1.01–1.40)	1.19*	(1.01–1.40)
Anyone hospitalized due to COVID-19			0.73	(0.47–1.14)	0.73	(0.47–1.14)
Anyone died due to COVID-19			1.20	(0.70–2.06)	1.20	(0.70–2.05)
Personal contacts at least once a week			0.76***	(0.66–0.88)	0.76***	(0.66–0.88)
Electronic contacts at least once a week			1.21*	(1.03–1.42)	1.21*	(1.03–1.42)
Deaths per 100,000					1.01	(1.00–1.02)
Days with stringency > 60					1.00	(0.99–1.01)
	Var.	CI	Var.	CI	Var.	CI
Intercept	0.14	(0.07–0.27)	0.16	(0.09–0.28)	0.12	(0.07–0.22)

AIC = Akaike Information Criterion, BIC = Bayesian Information Criterion, OR = odds ratio, CI = 95%-confidence interval, Var. = variance component,

*Ref.* = reference category, *** *p* < 0.001, ** *p* < 0.01, * *p* < 0.05

#### Fixed predictors with random intercepts

At the individual level, respondents who are living alone have a significantly higher probability of feeling lonelier since the outbreak of the pandemic (OR = 2.19; 95%-KI = [1.72–2.80]). Personal contacts at least once a week reduce the probability of feeling lonelier (OR = 0.76; 95%-KI = [0.66–0.88]), whereas electronic contacts at least once a week (OR = 1.21; 95%-KI = [1.03–1.42]) increase feelings of loneliness since the outbreak of the pandemic. Age is not significantly associated with the outcome variable. At the macro-level, neither the number of cumulated deaths nor the number of days with a stringency index above 60 is associated with feeling lonelier since the pandemic started. The model with fixed predictors and random intercepts improves compared to the intercept-only model (AIC = 19,914.72; BIC = 20,062.97). When including the variables at the macro-level, the goodness-of-fit measures indicate that the model does not further improve (AIC = 19,912.82; BIC = 20,077.54). When comparing the multilevel model with random intercepts at individual level with a model with fixed intercepts at individual level, there were only marginal differences regarding two control variables and no differences regarding the predictors.

#### AMEs of interactions with exploratory variables at individual level with macro-variables

**Fig. 6:**
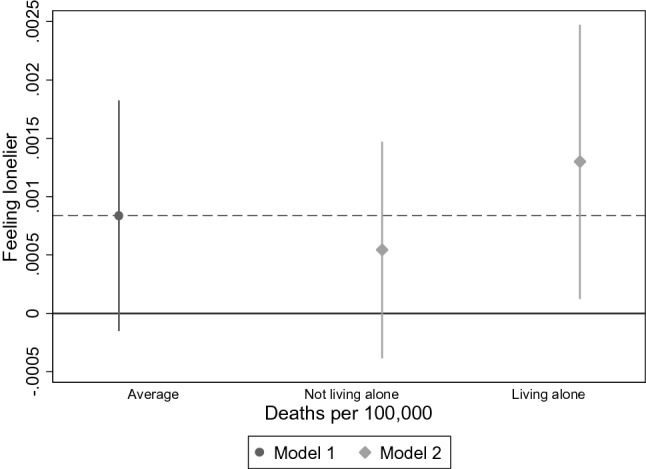
Average marginal effects of main and interaction effects for feeling lonelier and number of deaths for living alone (*n* = 27,889). *Model 1* = *Main effect of deaths per 100,000, Model 2* = *interaction with number of deaths and living alone*. AMEs were calculated for the model with fixed predictors at individual and macro-level with random intercepts, Data: Preliminary SHARE Wave 8 Release 0

Figure 6 shows the AME for number of deaths on feeling lonelier for living alone and the effects conditional on living arrangements. While the feeling of loneliness does not increase significantly with the number of deaths on average, including an interaction reveals that people who live alone feel lonelier. With rising mortality due to COVID-19, those living alone have a higher probability for increased feelings of loneliness.

**Fig. 7:**
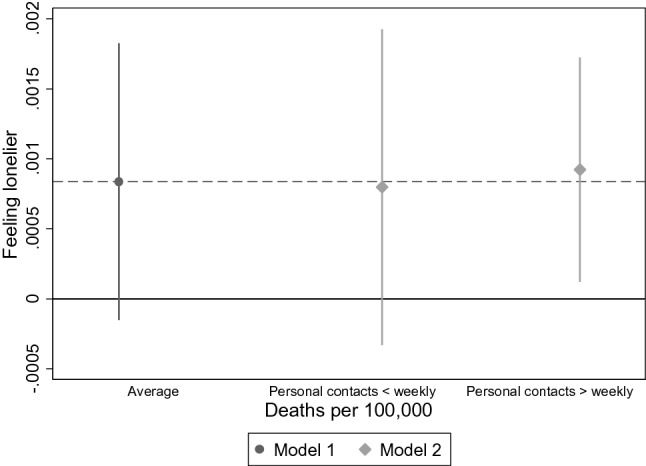
Average marginal effects of main and interaction effects for feeling lonelier and number of deaths for personal contacts (*n* = 27,889). *Model 1* = *Main effect of deaths per 100,000, Model 2* = *interaction with number of deaths and personal contacts*. AMEs were calculated for the model with fixed predictors at individual and macro-level with random intercepts, Data: Preliminary SHARE Wave 8 Release 0

Figure 7 further suggests the relationship between number of deaths and feeling lonelier being moderated by frequency of personal contacts. While feelings of loneliness are on average not significantly increasing with number of deaths, they are for those with more frequent personal contacts. However, this is largely due to greater precision in the estimate for those with frequent contacts, the difference between groups is not significant. All other interaction effects were not significant (see Supplementary Material).

## Discussion

Based on data from the first SHARE Corona Survey, this study sheds light on the mental well-being of retired individuals aged 60 plus. Data were collected between June and August 2020, which is a specific period because the first wave of the COVID-19 pandemic subsided at that time in most countries. Additionally, it should be highlighted that the results for the retired population aged 60 and above cannot be generalized for other parts of the population. Furthermore, the results cannot be interpreted causally but rather as correlations. Finally, the number of entities at the 2nd level was lower than the usual recommendation for multilevel models which might lead to biased standard errors (Maas & Hox 2005). Including the regional level in the multilevel models would add additional variation because the first wave of the pandemic hit regions within countries with different timing and intensity and would increase the number of entities at the 2nd level. However, this was not possible due to a lack of available sources for regional data in all 26 countries. Nevertheless, our study belongs to the first ones that focuses the mental well-being of older adults in a cross-national setting. Using multilevel models with random intercept enables us to integrate both individual and macro-factors at country level. The validity of the random effects assumption in our application for the individual level variables is supported by robustness checks, which produce the same results in the models with random or fixed country level intercepts.

On a descriptive level, we observe huge differences between countries regarding the prevalence of increased feelings of sadness/depression and loneliness. For a large part of those who reported sadness/depression or loneliness, the situation worsened after the outbreak of the pandemic. This supports the conclusion formulated by Groarke et al. (2020) that supportive interventions to reduce negative well-being consequences should focus on those with mental health symptoms. Additionally, descriptive country comparative analyses reveal that there is a considerable association between the country differences in the prevalence of feeling more depressed and both the number of deaths and the number of days with stringent epidemic control measures.

The results of the multivariate multilevel regression models show that the influence of both macro-variables on increased feelings of loneliness is insignificant. However, both macro-indicators have an impact on the increase in depressive feelings. Both one additional death per 100.000 inhabitants and one additional day with a stringency index above 60 lead to a statistically significant increase in the probability for increased depressive feelings. A general trend which of the two macro-indicators is more influential is not apparent in the models. Future studies could focus on the question which role the timing might play (e.g., regarding the implementation of specific epidemic control measures) and whether other factors at macro-level could help to explain differences regarding the well-being consequences of the pandemic.

The multilevel models further show that, on individual level, age is not significantly associated with feeling more depressed or lonelier since the outbreak of the pandemic. However, in countries with higher death rates and with a larger number of days with stringent measures, the elderly (aged 80 and over) have an increased likelihood for feeling more depressed. Those living alone, especially in countries with high mortality rates due to COVID-19, are most at risk for feeling lonelier. Personal contact at least once a week has a positive influence on mental well-being. The results support our last hypothesis that electronic contacts do not compensate for the loss of personal contacts and might even have a negative influence. Additional sensitivity analyses (not presented) show that this is particularly the case if respondents report having personal contacts less than weekly. Further research is needed regarding this finding and whether it only holds for seniors or for younger cohorts as well. However, a possible explanation that we cannot rule out might be self-selection: depressed and/or lonely people may particularly suffer from deprivation of personal contacts and thus switch to electronic contacts instead.

Further research is also needed in order to identify the groups of society at particular risk of suffering from decreased mental well-being as a consequence of the pandemic. Based on our findings, the elderly in countries with high death rates and stringent measures as well as older adults living alone are those at increased risk of feeling depressed or lonely. Interventions among those at-risk groups are needed to minimize the negative mental health consequences of the pandemic, which should be implemented by social policies. Preventative measures for increasing mental well-being could focus on enhancing the awareness of the personal ability to enhance one’s resilience (e.g., by changing their focus to things that are under one’s personal control instead of being overwhelmed by the unpredictable situation). Staying personally connected with friends and family can improve mental well-being. However, socializing via electronic contact does not seem to have a protective function for older age groups. Since mental health problems are often stigmatized (Conner et al. 2010; Graham et al. 2003), especially the older population should be encouraged to reach out for help or to seek mental health treatment if necessary. On the supply side, this entails facilitating access to psychological and psychiatric support for older people.

## Supplementary Information

Below is the link to the electronic supplementary material.

Supplementary file1 (DOCX 135 KB)
