# Supplementary material for: Depression and loneliness of older adults in Europe and Israel after the first wave of covid-19
Source: Eur J Ageing. 2021 Aug 24;19(4):849–61. doi: 10.1007/s10433-021-00640-8 (PMC8383247; doi:10.1007/s10433-021-00640-8)
Supplement: Supplementary file 1 — Supplementary file1 (DOCX 135 KB) [file 10433_2021_640_MOESM1_ESM.docx]

# Supplementary

S1

*Note*. Average marginal effects of main and interaction effects for feeling sadder/ more depressed and number of days with stringency above 60 for living alone (n = 27,889). *Model 1 = Main effect of number of days with stringency above 60, Model 2 = interaction with number of days with stringency above 60 and living alone*. AMEs were calculated for the model with fixed predictors at individual and macro level with random intercepts, Data: Preliminary SHARE Wave 8 Release 0.

S2

*Note*. Average marginal effects of main and interaction effects for feeling sadder/ more depressed and number of deaths for living alone (n = 27,889). *Model 1 = Main effect of deaths per 100,000, Model 2 = interaction with number of deaths and living alone*. AMEs were calculated for the model with fixed predictors at individual and macro level with random intercepts, Data: Preliminary SHARE Wave 8 Release 0.

S3

*Note*. Average marginal effects of main and interaction effects for feeling sadder/ more depressed and number of days with stringency above 60 for personal contacts (n = 27,889). *Model 1 = Main effect of number of days with stringency above 60, Model 2 = interaction with number of days with stringency above 60 and personal contacts*. AMEs were calculated for the model with fixed predictors at individual and macro level with random intercepts, Data: Preliminary SHARE Wave 8 Release 0.

S4

*Note*. Average marginal effects of main and interaction effects for feeling sadder/ more depressed and number of deaths for personal contacts (n = 27,889). *Model 1 = Main effect of deaths per 100,000, Model 2 = interaction with number of deaths and personal contacts*. AMEs were calculated for the model with fixed predictors at individual and macro level with random intercepts, Data: Preliminary SHARE Wave 8 Release 0.

S 5

*Note*. Average marginal effects of main and interaction effects for feeling sadder/ more depressed and number of days with stringency above 60 for electronic contacts (n = 27,889). *Model 1 = Main effect of number of days with stringency above 60, Model 2 = interaction with number of days with stringency above 60 and electronic contacts*. AMEs were calculated for the model with fixed predictors at individual and macro level with random intercepts, Data: Preliminary SHARE Wave 8 Release 0.

S 6

*Note*. Average marginal effects of main and interaction effects for feeling sadder/ more depressed and number of deaths for electronic contacts (n = 27,889). *Model 1 = Main effect of deaths per 100,000, Model 2 = interaction with number of deaths and electronic contacts*. AMEs were calculated for the model with fixed predictors at individual and macro level with random intercepts, Data: Preliminary SHARE Wave 8 Release 0.

S 7

*Note*. Average marginal effects of main and interaction effects for feeling lonelier and number of days with stringency above 60 for each age group (n = 27,889). *Model 1 = Main effect of number of days with stringency above 60, Model 2 = interaction with number of days with stringency above 60 and age*. AMEs were calculated for the model with fixed predictors at individual and macro level with random intercepts, Data: Preliminary SHARE Wave 8 Release 0.

S 8


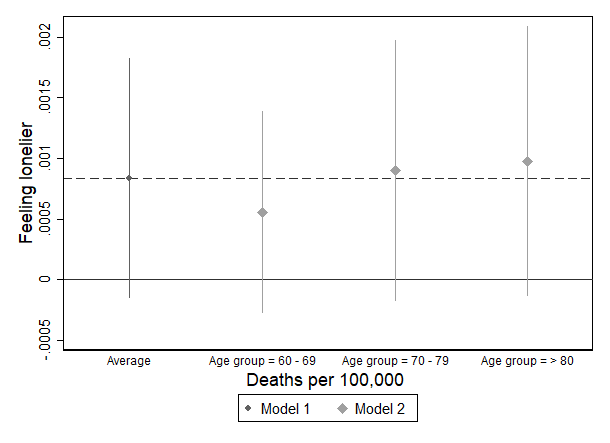


*Note*. Average marginal effects of main and interaction effects for feeling lonelier and number of deaths for each age group (n = 27,889). *Model 1 = Main effect of deaths per 100,000, Model 2 = interaction with number of deaths and age*. AMEs were calculated for the model with fixed predictors at individual and macro level with random intercepts, Data: Preliminary SHARE Wave 8 Release 0.

S 9


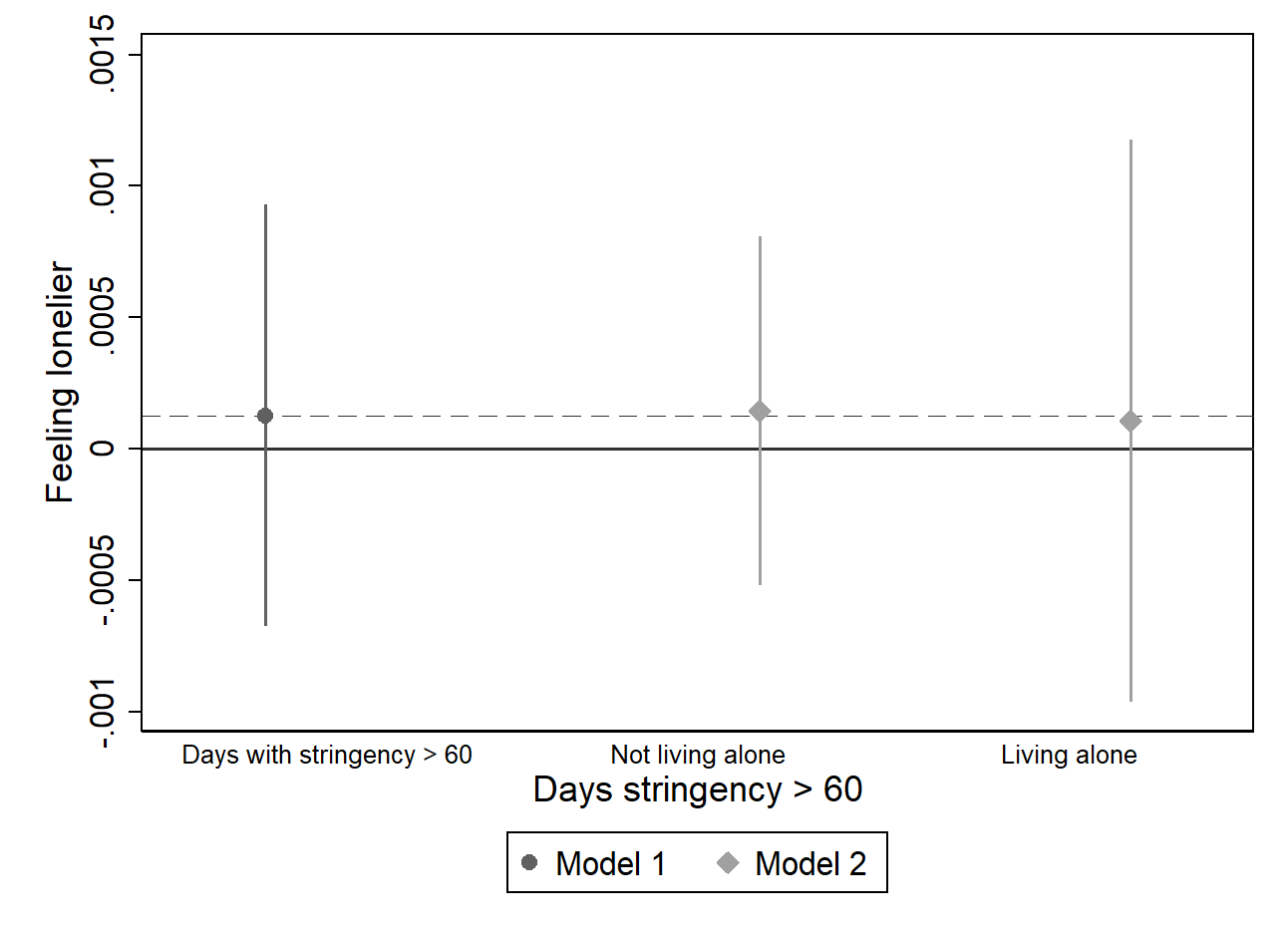


*Note*. Average marginal effects of main and interaction effects for feeling lonelier and number of days with stringency above 60 for living alone (n = 27,889). *Model 1 = Main effect of number of days with stringency above 60, Model 2 = interaction with number of days with stringency above 60 and living alone*. AMEs were calculated for the model with fixed predictors at individual and macro level with random intercepts, Data: Preliminary SHARE Wave 8 Release 0.

S 10


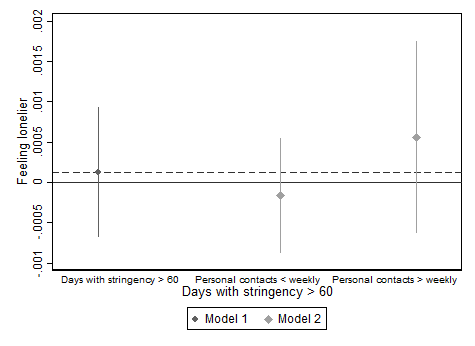


*Note*. Average marginal effects of main and interaction effects for feeling lonelier and number of days with stringency above 60 for personal contacts (n = 27,889). *Model 1 = Main effect of number of days with stringency above 60, Model 2 = interaction with number of days with stringency above 60 and personal contacts*. AMEs were calculated for the model with fixed predictors at individual and macro level with random intercepts, Data: Preliminary SHARE Wave 8 Release 0.

S 11

*Note.* Average marginal effects of main and interaction effects for feeling lonelier and number of days with stringency above 60 for electronic contacts (n = 27,889). *Model 1 = Main effect of number of days with stringency above 60, Model 2 = interaction with number of days with stringency above 60 and electronic contacts*. AMEs were calculated for the model with fixed predictors at individual and macro level with random intercepts, Data: Preliminary SHARE Wave 8 Release 0.

S 12


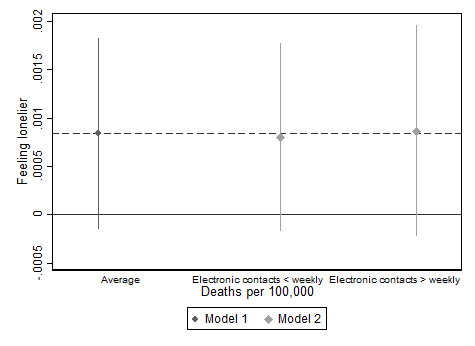


*Note.* Average marginal effects of main and interaction effects for feeling lonelier and number of deaths for electronic contacts (n = 27,889). *Model 1 = Main effect of deaths per 100,000, Model 2 = interaction with number of deaths and electronic contacts*. AMEs were calculated for the model with fixed predictors at individual and macro level with random intercepts, Data: Preliminary SHARE Wave 8 Release 0.
